# Infestation dynamics of *Triatoma dimidiata* in highly deforested tropical dry forest regions of Guatemala

**DOI:** 10.1590/0074-02760200203

**Published:** 2020-10-28

**Authors:** Daniel Penados, José Pineda, Michelle Catalan, Miguel Avila, Lori Stevens, Emmanuel Agreda, Carlota Monroy

**Affiliations:** 1 Universidad de San Carlos de Guatemala, Laboratorio de Entomología Aplicada y Parasitología, Ciudad de Guatemala, Guatemala; 2 Universidad de San Carlos de Guatemala, Facultad de Ciencias Químicas y Farmacia, Escuela de Biología, Ciudad de Guatemala, Guatemala; 3 Universidad de San Carlos de Guatemala, Centros de Estudios Conservacionistas, Ciudad de Guatemala, Guatemala; 4 University of Vermont, Department of Biology, Burlington, VT, USA

**Keywords:** Chagas disease, vector control, reinfestation, colonization, deforestation

## Abstract

**BACKGROUND:**

Deforestation, driven by anthropogenic change in land use, influences the behaviour and abundance of vector-borne diseases. For various species of Chagas disease vectors, there is evidence that change in land use affects population density and abundance. *Triatoma dimidiata* is the most important Chagas vector in Guatemala, and at least one million people live in *T. dimidiata* endemic areas; however, infestation dynamics vary among regions, from high infestation with all life stages to low seasonal infestation by sylvatic adults.

**OBJECTIVES:**

The aim of this study was to evaluate how land-use, combined with domiciliary risk factors, influences the infestation dynamics of *T. dimidiata* for four villages in a dry forest region with a strong deforestation history.

**METHODS:**

Land use, measured with drone and satellite images, was classified into four categories (houses, monocultures and pastures, woodland and shrubland, and bare soil). Domiciliary risk factors and infestation were assessed through entomological surveys. Statistical analyses compared infestation indices and the ability of land use and domiciliary risk factors to explain infestation.

**FINDINGS:**

Two villages had significantly higher infestation (26 and 30% vs. 5 and 6%), yet all villages had high colonisation (71-100% of infested houses had immature insects), with no significant difference among them. Because of the high level of deforestation across the study area, land use was not related to infestation; however, domiciliary risk factors were. A model based on four weighted domiciliary risk factors (*adobe* or *bajareque* walls, intradomicile animals, intradomicile clutter, and dirt floors) explains the infestation risk.

**MAIN CONCLUSIONS:**

Because almost all infested houses have reproducing populations in this deforested dry forest region and statistical analysis identified the domiciliary risk factors for infestation, intermediate and long-term control of Chagas disease vectors in this region requires management of these risk factors.

Deforestation driven by anthropogenic change in land use influences the behaviour and abundance of vector-borne disease.^1^ For example, disturbed habitats are associated with increased vector abundance compared with relatively undisturbed habitats for *Rhodnius pallescens,* the main vector of Chagas disease in Panama.^2^ In addition, high human population density, rain forest destruction, and human predation on local wildlife are associated with higher human *Trypanosoma cruzi* infections from the vectors *Rhodnius pictipes* and *Rhodnius neglectus* in Brazil.^3^

*Triatoma dimidiata* is the most important Chagas vector in Guatemala with at least one million people living in *T. dimidiata* endemic areas.^4^ Infestation dynamics of this vector vary regionally from high incidence of domestic reproducing populations as evidenced by all life stages throughout the year to low seasonal infestation by sylvatic adults.^5^ ^,^ ^6^ Seasonal infestation varies geographically with greater movement in April-June in Yucatan, Mexico,^7^ April-May in Costa Rica^8^ and March-May in Guatemala.^9^

Spraying with insecticides has been the most commonly used control mechanism across Chagas endemic regions, but relatively quick reinfestation (in as little as six-12 months for *T. dimidiata*), make control solely by insecticide problematic.^10^ ^,^ ^11^ Just as with infestation, reinfestation dynamics vary regionally. In Guatemala, reinfestation is less of a problem in the department of Zacapa,^11^ where reinfestation patterns reflect the vector’s seasonal movements from sylvatic populations.^12^ However, more recently, it has been shown the year round reinfestation in the dry forest region of the department of Jutiapa is driven by vectors that survive insecticide control treatments in nearby houses.^13^

The infestation and reinfestation patterns of *T. dimidiata* are influenced by both ecological and socioeconomic variables.^14^ As a consequence, for effective disease control, the variables contributing to domiciliary infestation, and thus influencing human epidemiology, need to be considered.^14^ ^,^ ^15^ This study evaluated land-use and domiciliary risk factors in the infestation patterns of *T. dimidiata* in four village in Jutiapa, Guatemala.

## MATERIALS AND METHODS

**Fig. 1:**
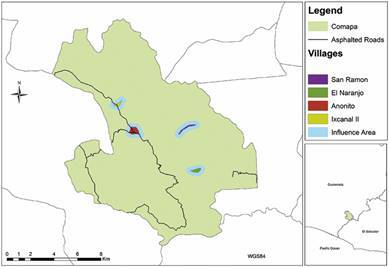
study area indicating the four villages within the municipality of Comapa, Jutiapa, Guatemala.

*Study area -* We worked in four villages (Anonito, Ixcanal II, San Ramon and El Naranjo) in the municipality of Comapa, department of Jutiapa, Guatemala. This study received ethical clearance from San Carlos University bioethics committee (AC-010-2018). Comapa, and indeed most of Jutiapa, is a Chagas endemic region, with a predominantly rural population. Monocultures of corn, beans and *jocote* (cashew, *Spondias purpurea*), along with cattle raising, are the major crops and drive the land use changes and loss of forest coverage. Of the four villages, Anonito [1,200-1,300 meters above sea level (masl)], has the most houses, with 209, followed by Ixcanal II (1,200-1,250 masl) and San Ramon (900-1,000 masl), with 153 and 109 houses, respectively, and, finally, El Naranjo (500-550 masl), with 62 houses. Anonito is also the largest village at 38.9 ha, followed by El Naranjo with 17.2 ha, San Ramon with 16 ha and Ixcanal II with 12.7 ha (Fig. 1). In all four villages, most of the houses are constructed with rustic mud-based materials, including *adobe* and *bajareque*, known to be high risk for *T. dimidiata* infestation. 

*Entomological survey* - Houses from each village were systematically searched in 2018 (Table I) by personnel specialised in vector control and trained in safe handling of vectors from the Ministry of Public Health and Social Assistance (MSPAS). A small number of houses in each village were uninhabited (2-11%) and not searched and in some cases a family chose not to participate (2-8%) (Table I). Searchers used the man-hour method, that is, using a flashlight or headlamp and forceps, one technician actively searched each house for vectors for one hour, starting with the intradomicile and following with the peridomicile. 

**TABLE I:** Number and percentage of houses surveyed and not surveyed in each village in 2018

Village	Surveyed n (%)	Uninhabited n (%)	Households not participating n (%)	Total houses n	Months surveyed
Anonito	181(86.6)	23 (11.0)	5 (2.4)	209	June
Ixcanal II	140 (91.5)	5 (3.3)	8 (5.2)	153	August
El Naranjo	52 (83.9)	5 (8.1)	5 (8.1)	62	September
San Ramon	104 (95.4)	2 (1.8)	3 (2.8)	109	September

All vectors found were collected and placed in mesh-covered plastic bottles labelled with the house number, name of the head of the household, date and ecotope (intra- or peridomicile). Within 15 hours, captured bugs were preserved individually in vials containing 95% ethanol and collection and demographic (sex or life stage) information was recorded in a notebook and an electronic database. Based on the entomological survey, we calculated frequently used entomological indices (Table II): infestation = (number of houses with vectors found/number of surveyed houses) × 100; colonisation = (number of houses with nymphs found/number of houses with vectors) × 100; intradomiciliary infestation = (number of houses with vectors in the intradomicile/number of surveyed houses) × 100; peridomiciliary infestation = (number of houses with vectors in the peridomicile/number of surveyed houses) × 100; overcrowding = (total number of vectors found in the village/number of houses with vectors); density = (total of vectors found in the village/number of surveyed houses). 

**TABLE II:** Infestation risk variables for *Triatoma dimidiata* infestation

Evaluated condition	Weight points
Cracks in rustic material walls like *adobe* or *bajareque*	3.5
Vector or vector’s signals present, e.g., faeces or exuviae	1.5
Presence of domestic animals in the intradomicile	1
Chicken coops in the intradomicile	1.5
Firewood in the intradomicile	0.5
Construction material in the intradomicile	0.5
House older than six years	0.5
Accumulated objects and disorder in the intradomicile	0.5
Dirt floor	0.5

Among villages, the infestation and colonisation were compared using a chi-square test, performed in the software R 3.6.3,^16^ with the function “chisq.test” in the Cran package “stats”.

*Land use quantification -* Land use was quantified using both drone and satellite image data. Drone data for each village included a 300 m influence area (village periphery likely to be a source of vectors) based on movement estimates of 45-60 m/15 days for non-domiciliated *T. dimidiate.* ^17^ Satellite data were assessed for the entire municipality of Comapa. 

Drone image data with resolution of 12 cm/pixel was obtained with a DJI Phantom 3 Advanced programed with Map Pilot 4.0 (Drones Made Easy, San Diego, USA), with altitude 300 m, speed 15 m/s, adjustable speed in shaded areas option and 70% overlap in the images. Drone images were assembled using the software Agisoft Photoscan 1.4.5 (St. Petersburg, Russia)^18^ to generate a photogrammetric mosaic of each village and its influence area. Land use coverage was classified with the Supervised Classification tool of ArcGis Pro 2.5 (ERSI, Redlands, USA).^19^ First, a cloud of points was generated identifying the most representative land use classes: (i) woodland and shrubland; (ii) monocultures; (iii) cattle pastures and bare soil; and (iv) houses. The Create Signatures tool was used to generate the mean values and a covariance matrix of the colours of each land use class. The Create Signatures output was used to classify maximum verisimilitude creating a raster classified with each land use class. Create Accuracy Assessment Points was used to assess raster accuracy with the sampling strategy fields “ground truth” and “stratified random”. Update Accuracy Assessment Points were inputs to Compute Confusion Matrix to estimate the expected accuracy value for each class > 80%.

Atmosphere corrected Sentinel 2B satellite images^20^ covering the entire municipality of Comapa from the month with lowest cloud coverage, December, for 2016, 2017 and 2018 (Copernicus Open Access Hub, https://scihub.copernicus.eu/), were classified in a similar manner. There were three classes for the satellite data: (i) monocultures and pastures; (ii) woodland and shrubland; and (iii) bare soil. 

*Domiciliary infestation risk -* While the personnel of MSPAS carried out the entomological survey at each house, personnel of the Applied Entomology and Parasitology Laboratory (LENAP) carried out a Capacities, Aptitudes and Practices (CAP) survey.^21^ For each surveyed household, socioeconomical, capacities, knowledge and customs information related to Chagas disease and *T. dimidiata* infestation was collected. A copy of the survey is included as Supplementary data.

Focusing on the village with highest infestation, Anonito, we modelled house infestation risk using variables from the CAP survey based on work by Bustamante et al.^9^ ^,^ ^21^ from a similar environment in Guatemala. Variables included were: cracks in walls constructed from rustic material; vector presence or sign of vector presence (exuvia or faeces); domestic animals in the domicile; chickens in the domicile; firewood in the domicile; construction material outside the house; house older than six years; accumulated objects inside the house; and dirt floor. The sum of the variables scored as presence/absence and weighted according to Bustamante et al.^9^ ^)^ (Table II) was the infestation risk, ranging from 0-10, with 0 being no infestation risk. 

*Statistical analysis of domiciliary infestation risk model -* The efficacy of the domiciliary infestation risk model to predict infestation risk as the number of vectors per house was evaluated with the Cran package “pscl” in using a zero - inflated model based on a Poisson distribution with the function “zeroinfl” (Fig. 4). This model assumes the sampling method underestimates the response variable,^22^ infested houses, and is a strong test because there were houses with traces of vector presence (exuviae or faeces), but in which no vectors were caught.

After developing the model with Anonito, it was evaluated for Anonito, Ixcanal II and San Ramon. El Naranjo (500 masl) although capable of maintaining *T. dimidiata* populations is outside the major distribution range (800-1,500 masl) of this vector^6^ and because only three houses were infested, it was not included. Kruskal-Wallis test was used to compare the risk values among the villages.

## RESULTS

Entomological

*Survey -* Across the four villages, the 80% of the vectors were founded in cracks in rustic material walls like *adobe* or *bajareque*. There were two patterns among the four villages for three of the six (infestation, intradomiciliary infestation and density) entomological indexes (Table III). Anonito and Ixcanal II presented high infestation indices, 36 and 27%, respectively, in contrast with El Naranjo and San Ramon, with 6 and 5%, respectively. In contrast, infestation in the peridomicile was low in all villages (0-4.23%). Colonisation indices were high overall (all > 70%). Although El Naranjo had relatively few vectors, it had the highest overcrowding with an average of 11 vectors per infested house, over twice that in the other villages (three-four vectors per house). Density values were similar to infestation, Anonito was highest for both (density = 1.6), followed by Ixcanal II (1.06), El Naranjo (0.64) and San Ramon (0.14). The chi-square test indicated significant differences (p < 0.005) in infestation among villages, but not in colonisation values (p > 0.95).

**TABLE III:** Entomological indices

Village	Infestation %	Colonisation %	Intradomiciliary infestation %	Peridomiciliary infestation %	Overcrowding	Density
Anonito	36.50	71.21	36.50	1.66	4.32	1.57
Ixcanal II	26.76	73.68	24.65	4.23	3.95	1.06
El Naranjo	6.00	100.00	6.00	0.00	10.67	0.64
San Ramón	4.95	100.00	3.96	1.00	2.8	0.14

Infestation = (number of houses with vectors found/number of surveyed houses) × 100; colonisation = (number of houses with nymphs found/number of houses with vectors) × 100; intradomiciliary infestation = (number of houses with vectors in the intradomicile/number of surveyed houses) × 100; peridomiciliary infestation = (number of houses with vectors in the peridomicile/number of surveyed houses) × 100; overcrowding = (total number of vectors found in the village/number of houses with vectors); density = (total of vectors found in the village/number of surveyed houses).

*Land use quantification* - For the four drone classification categories evaluated at each village and influence area - (i) woodland and shrubland; (ii) monocultures; (iii) cattle pastures and bare soil; and (iv) houses -, we founded San Ramon had the highest woodland and shrubland (60%), followed by El Naranjo (58%), Anonito (55%) and Ixcanal II (53%) (Fig. 3). San Ramon had the lowest in two categories, monocultures and cattle pastures and bare soil, followed by Anonito and El Naranjo, while Ixcanal II was highest in these categories.

Based on the three satellite image categories evaluated for the entire municipality of Comapa - (i) monocultures and pastures; (ii) woodland and shrubland; and (iii) bare soil -, the largest category was monocultures and pastures, being relatively constant at ~58% over the three years (Fig. 4). The intermediate category, woodland and shrubland, decreased from ~32% in 2016 to about 25% in 2017 and 2018. Finally, bare soil was the least abundant category, but increased from 10% in 2016 to about 18% in 2017 and 2018. Qualitatively, the percentage of bare soil seemed to increase from north to south (Fig. 4). The monocultures and pastures were concentrated in the northern and southern regions, whereas the woodland and shrubland were distributed primarily in the center.

Over the three years, more that 55% of Comapa was monocultures and pastures, and the more natural vegetation of woodland and shrubland decreased over the three years, being replace by bare soil. Overall, there was an increase from 65 to 75% of the study area with monoculture, pastures and bare soil and a decrease from 32 to 25% of more natural vegetation of woodland and shrubland.

**Fig. 2:**
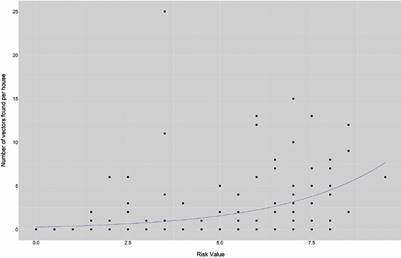
domiciliary infestation risk model using weighted variables from the Capacities, Aptitudes, and Practices (CAP) survey. The zero - inflated model, based on a Poisson distribution showed a significant, positive correlation (p < 0.01).

*Statistical analysis of domiciliary infestation risk model -* The risk model showed a positive and significant (p < 0.01) correlation between infestation risk and the number of vectors collected per house (Fig. 2). In addition, the model was exponential with the vector presence strongly increasing around an infestation risk of 2.5.

**Fig. 3:**
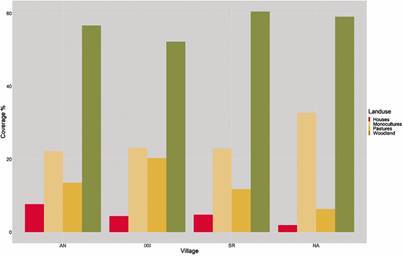
drone image analysis of land use percentage for four categories - (i) woodland and shrubland; (ii) monocultures; (iii) cattle pastures and bare soil; and (iv) houses -, for four villages, Anonito (AN), Ixcanal II (IXII), San Ramon (SR) and El Naranjo (NA), in Comapa, Jutiapa, Guatemala.

**Fig. 4:**
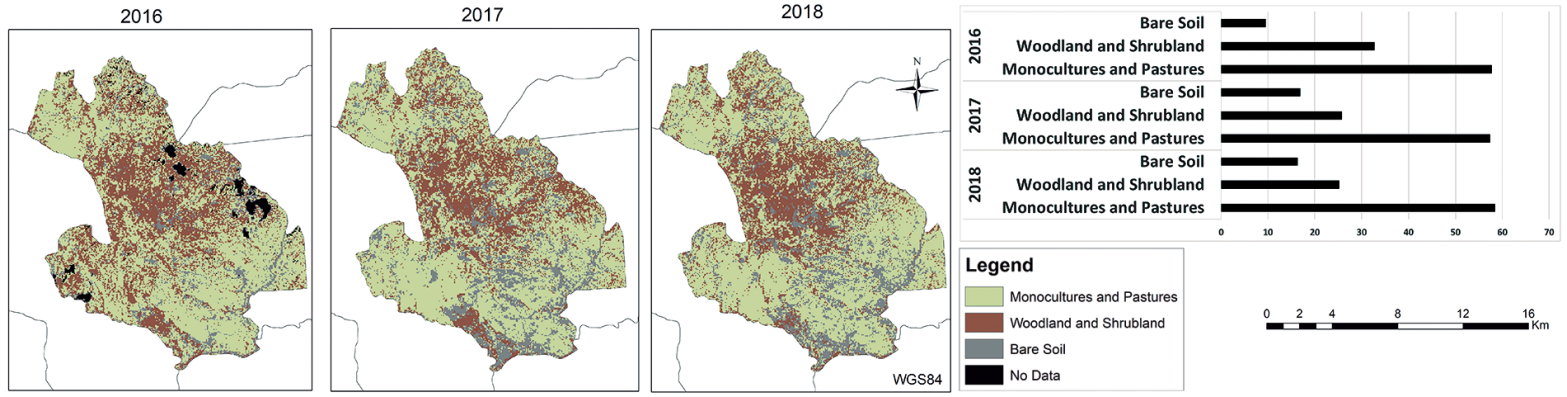
satellite image analysis of land use for three categories - (i) monocultures and pastures; (ii) woodland and shrubland; and (iii) bare soil -, for Comapa, Jutiapa, Guatemala. Data are from December 2016, 2017 and 2018.

Comparing the infestation risk among villages showed Ixcanal II and El Naranjo similar (mean ~7) and close to the third quartile. Anonito had mean infestation ~4.5 and in the centre of the distribution. Lastly, San Ramon had the lowest infestation risk ~2.5 and closer to the first quartile. The non-parametric Kruskall-Wallis test indicated that San Ramon had a lower infestation risk in comparison to the other villages (p < 0.005).

## DISCUSSION

The four villages showed two patterns, Anonito and Ixcanal II, had significantly higher infestation indices (25-35%), indicating high infection risk for the human population, whereas San Ramon and El Naranjo had low infestation indices (< 8%), below the threshold of infestation for transmission risk.^23^ This suggests that the villages differ in factors that contribute to the *T. dimidiata* infestation risk.^2^ ^,^ ^14^

The high colonisation indices (70-100% of infested houses had nymphs) showing active reproduction of the vector, indicate that the vector population is highly adapted to houses.^7^ ^,^ ^24^ The high rate of intradomicile infestation compared to peridomiciliary infestation further supports that the vector population is highly adapted to intradomiciliary variables such as cracks in the walls, clutter, accumulated objects and animals sleeping inside the house.^14^ Vector control programs have aimed to reduce colonisation by Chagas disease vectors by addressing these variables. A highly infested village represents many houses with infestation risk factors and a vector population well adapted to the domicile all combining to increase the epidemiological risk of the human population.^15^ ^,^ ^24^

The mean of 10 bugs per infested house (overcrowding index) in one village (El Naranjo) was over twice that (3-4) in the other three villages. El Naranjo had few infested houses, but the vectors were highly concentrated in the few infested houses. Thus, the people living in infested houses in El Naranjo could be at a higher risk than people living in infested houses in the other villages. Lastly, the higher densities observed in Anonito and Ixcanal II indicated more homogeneously distributed populations.

Examining land use, the four villages had similar woodland and shrubland coverage, but different infestation indices. We had expected the opposite, more woodland and shrubland in the less infested villages. This discrepancy could be because the local populations of bugs are highly adapted to domiciliary conditions as discussed above.^14^ ^,^ ^24^ For these village in Comapa, Jutiapa, the variation in forest coverage has no influence on infestation and reinfestation patterns, and it has been shown that resistant or well-hidden vectors surviving insecticide treatment drive the reinfestation patterns.^13^ This contrasts with in other areas, for examples Colombia, where forest coverage is an important risk factor for infestation, which is largely driven by wild sylvatic populations.^12^

Land use in Comapa was shown by satellite image analysis to be mostly monocultures and pastures, the amount of bare soil increased between 2016 and 2017-2018 and the amount of woodland and shrubland decreased. This is consistent with the strong history of deforestation that has removed most of the wild populations of vectors in the region and reflected in the main economic activities, cattle raising and monoculture crops, both of which are implemented in destruction of the dry tropical forest of Central America.^25^ It has been demonstrated previously that areas with very degraded forests have little or no wild triatomine presence.^26^ A drastic reduction of the *T. cruzi* infection in wild mammals, such as opossum, is also an indication of the absence of triatomines in very deforested regions.^27^ This suggests that, in our study area, a tropical dry forest region with a history of strong deforestation driven by economic activities like monocultures and cattle pastures, the forest coverage does not currently influence the infestation and reinfestation patterns of *T. dimidiata* because the degradation and deforestation have reached a point where there are no surviving wild vector populations. Cahan et al.,^13^ in an ecologically similar area in Jutiapa, with villages surrounded by deforested dry forest, demonstrated with genomic data that reinfestating vectors came predominantly from nearby domiciliary resilient populations, diminishing the importance of wild vector populations in the reinfestation dynamics. Cahan’s study documents the importance of managing variables that sustain infestation and colonisation to effectively control the Chagas transmission by *T. dimidiate.* ^13^

The village level drone analysis indicated high (52-60%) woodland coverage, yet regional satellite analysis indicated lower (25-32%) woodland and shrubland coverage. The difference may be due to community practices where large trees are conserved in a village and its near surroundings. The area covered by the canopy of these large village trees was classified as woodland in drone images. In contrast, the satellite images analysed on a larger spatial scale, highlight the main economic activities, monocultures and cattle grazing, that occur further away from the villages. Drone flights generate detailed information over smaller areas, but in this scenario, the satellite images clearly capture the land-use dynamics across the region of Comapa, Jutiapa, resulting in a better approach to understand *T. dimidiata* vector dynamics.

**Fig. 5:**
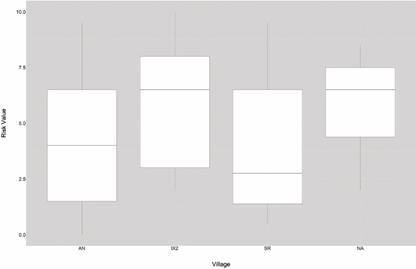
average domiciliary risk for each of the villages Anonito (AN), Ixcanal II (IX2), San Ramon (SR) and El Naranjo (NA). Kruskal-Wallis test indicated a significantly lower risk in San Ramon (p < 0.005).

The weighted variables used to estimate the domiciliary infestation risk (Table II) showed a positive and significant correlation with the number of vectors found in a house (Fig. 5). That these variables increased domiciliary infestation risk has been shown in multiple studies in Jutiapa.^9^ ^,^ ^14^ ^,^ ^15^ ^,^ ^28^ Additionally, it has been documented that mitigating these risk factors decreases infestation and colonisation by *T. dimidiate.* ^15^ ^,^ ^21^ ^,^ ^28^ The most important variable was cracks in the walls, which provide hiding places for the vectors, and where 80% of the vectors were found in this study. This finding supports that *adobe* or *bajareque* construction was not the driving factor, but rather associated variables such as wall cracks and dirt floor are. In all four villages, rustic materials were the main housing construction materials, but in San Ramon the walls were in better condition with fewer cracks, leading to a lower infestation.

Our analysis of the weighted variables showed that the model is efficient at determining the risk of a house being infested in highly deforested dry forest regions. In addition, the risk model shows a similar pattern for the infestation index and density variables (data not shown). Anonito and Ixcanal II had higher entomological indices and a higher proportion of high-risk houses. In contrast, San Ramon had a lower infestation and density, and also a lower proportion of high-risk houses. El Naranjo had the highest proportion of high-risk houses, but a low infestation index. This was probably due to the altitudinal distribution of *T. dimidiata*; most vectors were found between 800-1,500 masl^6^ although they can persist as low as 500 masl, the altitude of El Naranjo, thus possibly explaining the low infestation there. 

We did not look for *T. dimidiata* in the villages’ influence area because current vector collection methods in sylvatic areas are complicated and have unreliable results due to low detectability. Such information would provide stronger insights on the influence of infestation from this area as compared to our assumption of the absence of an influence due to the lack of data. However genetic analysis of relatedness within the vector population^29^ has also shown the vector to be quite mobile. 

Because of the high deforestation throughout the study area and subsequent lack of data from *T. dimidiata* endemic regions that are not deforested, we understand that our data does not generate strong evidence for the role of deforestation in the conclusion that highly deforested dry forest regions generate a pattern of highly colonised villages. Nonetheless, this study is the first to present evidence that, is in villages with low infestation, 100% of houses have reproducing populations of T. dimidiata that, in highly deforested dry forest regions, T. dimidiata populations are well adapted to domiciliary habitats. A broader analysis, using satellite images, and including more diverse land use patterns, could clarify the effect of deforestation in the domiciliation of *T. dimidiata*. Because deforestation is pervasive throughout the study region, and in fact throughout much of Central America, differences in infestation among these villages may not reflect variation in land use such as woodland coverage, but rather domiciliary variables such as cracks in *adobe* and *bajareque* walls, animal presence, clutter within the house and a dirt floor. These variables have been consistently used to assess infestation. 

This study presents evidence that in highly deforested dry forest regions, *T. dimidiata* populations are well adapted to domiciliary habitats, which results in a higher transmission risk for human populations. A nearby forest area could help mitigate future reinfestations after insecticide treatment.^15^ Combined with previous studies^13^ ^,^ ^29^ in the department of Jutiapa, there is consistent evidence that in highly deforested dry forest regions with vector populations well adapted to the houses following insecticide spraying a subsequent infestation of a domiciliary resilient population is observed.^13^ For vector control purposes, the resilient domesticated population of *T. dimidiata* could be tackled by implementing house improvements that reduce infestation risk, such as plastering cracks in the walls, replacing the dirt floor and removing animals from inside houses.^15^ ^,^ ^28^ Given these observed patterns of highly adapted populations in degraded and deforested dry forest regions, we conclude that it is critical to manage the domiciliary variables that controlled infestation for immediate and long-term control of Chagas diseases vector transmission in the region.
